# Characteristics of stepfamilies and maternal mental health compared with non-stepfamilies in Japan

**DOI:** 10.1186/s12199-017-0658-z

**Published:** 2017-05-18

**Authors:** Masako Sugimoto, Yoshie Yokoyama

**Affiliations:** Department of Public Health Nursing, Osaka City University, 1-5-17 Asahi-machi, Abeno-ku, Osaka, 545-0051 Japan

**Keywords:** Stepfamily, Mother, Depression

## Abstract

**Background:**

Stepfamilies remain poorly understood in Japanese society, and the support needs of stepfamily mothers are unclear. This study aimed to identify characteristics of stepfamilies and maternal mental health as compared with non-stepfamilies in Japan to utilize as a primary resource for providing effective support through community-based health care for stepfamilies.

**Methods:**

From December 2011 to July 2012, we conducted this questionnaire survey with mothers at 3- and 4-month checkups for infants. The response rate was 75.1%. The sample for analysis included responses of 2246 mothers, excluding single mothers.

**Results:**

Respondents comprised 47 (2.1%) stepfamilies and 2199 (97.9%) non-stepfamilies. There were significantly higher rates of parents with not more than a high school education and ≥3 children among stepfamilies compared with non-stepfamilies. Stepfamily mothers had significantly higher rates of feeling a lack of economic resources, absence of participation in childbirth education classes, smoking during pregnancy, and unplanned pregnancy. Furthermore, they also had significantly higher rates of depression and a lack of confidence in the parent role. Maternal depression was associated with factors such as maternal age, self-perceived health, stress level, confidence in breastfeeding, confidence in the parent role, and number of children.

**Conclusions:**

These findings suggest that stepfamilies exhibit many characteristics related to social disadvantage and problems with community-based health care in Japan. Healthcare providers should be aware of stepfamily mothers’ support needs and should put in place a support system for stepfamilies. Moreover, compared with non-stepfamily mothers, stepfamily mothers have a significantly higher prevalence of depression. However, stepfamily composition does not necessarily increase the risk of maternal depression. Therefore, healthcare providers should put in place a system for obtaining more thorough information about stepfamilies and conduct an early assessment to identify their support needs.

## Background

According to the Ministry of Health, Labour and Welfare [1], Japan had 222,107 divorces, with an incidence rate of 1.77 per 1000 population. The divorce rate in Japan is still lower than that in Western countries but has trended upward since the 1990s. In addition, 58.4% of these divorces involved dependent children. Whereas approximately 120,000 children experienced a parental divorce in 1975, approximately 220,000 did so in 2014. Since the latter half of the 1990s, every year more than 200,000 children experienced a parental divorce in Japan [1]. Meanwhile, the rate of remarriage has increased along with Japan’s increasing divorce rate. In 1975, 12.7% of marriages involved at least one partner who had been married previously compared with 26.4% in 2014 [1]. Therefore, approximately one out of every four new marriages is remarriages, giving rise to various family forms, such as stepfamilies, as seen in Japan today.

Ganong and Coleman broadly defined a stepfamily as a family “in which at least one of the adults has a child (or children) from a previous relationship” [2]. However, although many stepfamilies are formed after the remarriage of one or both partners, that is not always the case [3]. We must also emphasize that stepfamily membership is not necessarily confined to those who are in the same household [2]. In the USA, the stepfamily was actualized socially after the 1970s when divorce and remarriage had increased and stepfamily research drastically increased after the 1980s [2, 4, 5]. In contrast, the stepfamily in Japan has received little attention socially and academically until recently [6]. However, stepfamily research in Japan has increased since 2000. Several studies on stepparents and biological parents have shown that difficulties during the family formation process can easily occur in stepfamilies, and particularly for stepmothers, who experience higher stress levels [5–8]. These findings accord with stepfamily research in Western societies [9–11]. In addition, several studies have shown that residing in a stepfamily is a risk factor for child abuse [12, 13]. Although these findings suggest the need to support mothers in stepfamilies, Japanese society has a poor understanding of the stepfamily, and even the need for support is not yet recognized [8]. Furthermore, no clear academic distinction has been drawn between stepfamilies and non-stepfamilies (i.e., families with both biological parents), and there are no population-based studies on stepfamilies. As a result, the support needs of stepfamily mothers remain unclear. Against this background, the purpose of this study was to identify characteristics of stepfamilies and maternal mental health as compared with non-stepfamilies. This information can be used as a primary resource for providing effective support through community-based health care for stepfamilies.

## Methods

### Study participants

This questionnaire survey was conducted with 3008 mothers at 3- and 4-month health checkups for infants in city A from December 2011 to July 2012. City A is located in an urban area composed of a residential community of around 480,000 people with yearly birth numbers of approximately 4500. The consultation rate for 3- and 4-month health checkups in city A is approximately 98%. Participants were 2258 mothers (response rate, 75.1%). We excluded 12 single parents from the analysis based on reports that single parents have a higher risk of mental health problems and life difficulties, including a high poverty rate [14, 15]. Therefore, the sample for analysis included responses of 2246 mothers.

We clearly informed participants in writing that their participation in the study was voluntary, that no disadvantage would occur if they did not cooperate with the study, and that the return of the anonymous self-administered questionnaire would be taken as consent to participate in the study. The study protocol was approved by the Ethics Committee of Osaka City University.

### Survey content

We adopted Ganong and Coleman’s broad definition of stepfamilies [2], which does not limit them to marriage and does not necessarily confine stepfamily membership to those who are in the same household. Whether someone was part of a stepfamily or not was assessed with the question “Is your family a stepfamily? (a stepfamily means a family that includes a non-related parent-child relationship by remarriage or other such relation).” In addition, for any stepfamily, we asked how long the current partners had lived together.

Other survey items were basic personal parental information (age, educational attainment level, employment status); current family information (family structure, number of children, age of children, presence or absence of any disorder in children); family’s financial situation, maternal pregnancy situation, and childbirth situation (presence or absence of participation in a childbirth education course, smoking status during pregnancy, planned or unplanned pregnancy, presence or absence of complicated birth); and the child’s situation at childbirth (gestational age at birth, birth weight). In addition, we examined maternal self-perceived health and maternal mental health (depression, stress level) and factors affecting maternal mental health, including views on child care (confidence in child care, confidence in breastfeeding, confidence in parent role, recognition of child maltreatment) and support from the current partner and family environment (marital relations, support from family environment).

To measure maternal depression, we used the Edinburgh Postnatal Depression Scale (EPDS) by Cox et al. [16]. The EPDS is one of the most widely used self-report instruments for screening postpartum depression. The 10-item EPDS provides quantitative assessment of postpartum depression, with total scores ranging from 0 to 30 points. In Japan, scores over 9 points indicate postpartum depression [17]. Cronbach’s alpha was 0.79.

We measured stress level using the Semantic Differential Method [18], a widely used graphical rating scale that measures the connotative meaning that concepts and objects evoke in individuals. In this study, respondents were asked to mark the most appropriate point on a line, which had bipolar answers “strongly disagree” and “strongly agree” at the ends. We then scored respondents’ marks from 0 to 10, with higher scores indicating higher reported levels of stress (hereinafter called “stress score”).

Maternal self-perceived health was measured on a 6-point scale (“very good” to “poor”). We measured childcare confidence, confidence in breast feeding, and confidence in the parent role by assessing the following statements on a 4-point scale (“agree” to “strongly disagree”): “I can take care of my child,” “I can breastfeed my child as I expect,” and “I fulfill the parent role well,” respectively. For recognition of child maltreatment, we asked respondents, “Have you ever wondered whether you might mistreat your child?” Those who answered “yes” were considered persons recognized to be potential perpetrators of child maltreatment.

To measure marital relations, we used the Marital Love Scale by Sugawara et al. [19]. This 10-item self-report instrument has confirmed reliability and validity for assessing love relations between couples. Total scores range from 10 to 70 points, with higher scores indicating higher levels of intimacy of a couple. Cronbach’s alpha was 0.79 (hereinafter called “marital relations score”).

To assess childcare support in the family environment, we assessed the statement “I have childcare available when I need it” on a 4-point scale (agree to strongly disagree). To assess financial situation, we examined maternal self-perceived capability by assessing the statement “My family is financially capable” on a 4-point scale (agree to strongly disagree).

### Statistical analysis

We investigated independence of qualitative variables using the chi-squared test or Fisher’s exact probability test and analyzed the significance of differences between means using the *t* test. If a quantitative variable was non-normally distributed, we used the Mann-Whitney *U* test. Additionally, to reveal factors associated with maternal depression, we performed forced-entry multiple logistic regression analysis with maternal depression as the dependent variable. Independent variables were presence or absence of stepfamily, as well as factors associated with postpartum depression that were pointed out in previous studies [20, 21] (age, maternal self-perceived health, stress scores, confidence in breastfeeding, confidence in parent role, marital relations scores, recognition of support from the family environment, planned or unplanned pregnancy, presence or absence of complicated birth, and recognition of financial capability, mother’s educational attainment level, and number of children).

For all analyses, we considered *p* < 0.05 to indicate statistical significance. All statistical analyses were performed using IBM SPSS ver. 22.0 for Windows (Japanese IBM Corporation, Tokyo, Japan).

## Results

This study included 47 (2.1%) stepfamilies and 2199 (97.9%) non-stepfamilies. In stepfamilies, the mean (SD) living-together period with the current partner was 4.0 (3.0) years (range, 10 months to 12 years).

Table 1 summarizes the comparison of basic personal parental information between stepfamilies and non-stepfamilies. The mean (SD) age of stepfamily mothers and non-stepfamily mothers was 32.6 (6.0) years (range, 21–43 years) and 32.4 (4.6) years (range, 18–47 years), respectively. The mean (SD) age of stepfamily fathers and non-stepfamily fathers was 34.4 (6.7) years (range, 22–50 years) and 34.1 (5.5) years (range, 18–61 years), respectively. The rate of parents with not more than a high school education was significantly higher among stepfamilies (66.0% of mothers, 59.1% of fathers) than among non-stepfamilies (15.1% of mothers, 20.3% of fathers).

**Table 1:** Basic personal parental information in stepfamilies and non-stepfamilies

	Stepfamilies *n* = 47 *n* (%)	Non-stepfamilies *n* = 2199 *n* (%)	*p* value
Mothers			
Age			
Mean (SD, range)	32.6 (6.0, 21–43)	32.4 (4.6, 18–47)	.826^c^
Educational attainment level			
Not more than a high school education	31 (66.0)	329 (15.1)	<.001^a^
More than junior college graduation	16 (34.0)	1845 (84.9)	
Employment			
Yes	28 (60.9)	1468 (67.4)	.350^a^
No	18 (39.1)	710 (32.6)	
Fathers			
Age			
Mean (SD, range)	34.4 (6.7, 22–50)	34.1 (5.5, 18–61)	.791^c^
Educational attainment level			
Not more than a high school education	26 (59.1)	424 (20.3)	<.001^a^
More than junior college graduation	18 (40.9)	1665 (79.7)	
Employment			
Yes	44 (100.0)	2.077 (98.9)	.485^b^
No	0 (0.0)	23 (1.1)	

No answers were excluded

^a^Chi-squared test

^b^Fisher’s exact probability test

^c^ *t* test

As shown in Table 2, the rate of families with ≥3 children was significantly higher among stepfamilies (51.1%) than among non-stepfamilies (9.8%). Furthermore, the rate of families with a first child aged ≥5 years was significantly higher among stepfamilies (71.7%) than among non-stepfamilies (14.2%). As for family financial situation, we observed a significantly higher rate of feeling a lack of economic security (feeling financially well off) among stepfamily mothers (60.9%) than among non-stepfamily mothers (40.3%).

**Table 2:** Current family information and family financial situation in stepfamilies and non-stepfamilies

	Stepfamilies *n* = 47 *n* (%)	Non-stepfamilies *n* = 2199 *n* (%)	*p* value
Number of children			
1	5 (10.6)	1122 (51.4)	
2	18 (38.3)	847 (38.8)	<.001^a^
≥3	24 (51.1)	214 (9.8)	
Age of the first child			
0–1	5 (10.9)	1165 (54.2)	
2–4	8 (17.4)	678 (31.6)	<.001^a^
≥5	33 (71.7)	306 (14.2)	
Presence or absence of disorders in children			
No	44 (93.6)	2002 (91.7)	.631^b^
Yes	3 (6.4)	182 (8.3)	
My family is financially capable.			
Agree or slightly agree	18 (39.1)	1293 (59.7)	.005^a^
Disagree or strongly disagree	28 (60.9)	873 (40.3)	

No answers were excluded

^a^Chi-squared test

^b^Fisher’s exact probability test

Table 3 compares maternal pregnancy situation, childbirth situation, and child situation at childbirth between stepfamilies and non-stepfamilies. The rate of mothers who never participated in a childbirth education course was significantly higher among stepfamily mothers (57.4%) than among non-stepfamily mothers (22.2%). We observed a significantly higher rate of smoking during pregnancy among stepfamily mothers (19.6%) than among non-stepfamily mothers (2.4%). There was a significantly higher rate of unplanned pregnancy among stepfamily mothers (54.3%) than among non-stepfamily mothers (33.1%).

**Table 3:** Maternal pregnancy situation, childbirth situation, and child situation at childbirth in stepfamilies and non-stepfamilies

	Stepfamilies *n* = 47 *n* (%)	Non-stepfamilies *n* = 2199 *n* (%)	*p* value
Maternal pregnancy situation and the childbirth situation			
Presence or absence of participation in childbirth education classes			
Yes	20 (42.6)	1700 (77.8)	<.001^a^
No	27 (57.4)	484 (22.2)	
Smoking during pregnancy			
No	37 (80.4)	2116 (97.6)	<.001^b^
Yes	9 (19.6)	51 (2.4)	
Planned pregnancy			
Yes	21 (45.7)	1453 (66.9)	.003^a^
No	25 (54.3)	720 (33.1)	
Complicated birth			
No	41 (87.2)	1987 (91.5)	.298^b^
Yes	6 (12.8)	184 (8.5)	
Child situation at childbirth			
Gestational age (weeks)			
≥37	41 (93.2)	1999 (94.4)	.722^b^
<37	3 (6.8)	118 (5.6)	
Birth weight			
≥2500 g	43 (93.5)	1987 (91.8)	.684^b^
<2500 g	3 (6.5)	177 (8.2)	

No answers were excluded

^a^Chi-squared test

^b^Fisher’s exact probability test

Maternal self-perceived health, maternal mental health, and factors affecting maternal mental health (e.g., childcare views and support from current partner and family surroundings) are compared between stepfamilies and non-stepfamilies in Table 4. No significant differences were observed for maternal self-perceived health and stress scores. However, a significantly higher rate of depression was noted among stepfamily mothers (24.4%) than among non-stepfamily mothers (12.3%). We also observed a significantly higher rate of a lack of confidence in the parent role among stepfamily mothers (34.8%) than among non-stepfamily mothers (15.9%).

**Table 4:** Maternal self-perceived health, maternal mental health and associated factors in stepfamilies and non-stepfamilies

	Stepfamilies *n* = 47 *n* (%)	Non-stepfamilies *n* = 2199 *n* (%)	*p* value
Maternal self-perceived health and maternal mental health			
Maternal self-perceived health			
Very good, good, or normal	46 (97.9)	2094 (96.7)	.650^b^
Not so good or poor	1 (2.1)	72 (3.3)	
Depression (EPDS score)			
No (≤8)	34 (75.6)	1871 (87.7)	.015^a^
Yes (≥9)	11 (24.4)	262 (12.3)	
Stress score			
median (25, 75, min-max)	3 (1, 5, 0–8)	3 (2, 5, 0–10)	.730^c^
Maternal views of child care			
I can take care of my child			
Agree or slightly agree	44 (97.8)	2155 (99.1)	.372^b^
Disagree or strongly disagree	1 (2.2)	20 (0.9)	
I can breastfeed my child as I expect			
Agree or slightly agree	36 (78.3)	1819 (83.7)	.327^a^
Disagree or strongly disagree	10 (21.7)	355 (16.3)	
I fulfill the parent role well			
Agree or slightly agree	30 (65.2)	1825 (84.1)	.001^a^
Disagree or strongly disagree	16 (34.8)	346 (15.9)	
Recognition of child maltreatment			
No	40 (88.9)	1962 (92.3)	.400^b^
Yes	5 (11.1)	164 (7.7)	
Support from current partner and family environment			
Marital relations score			
median (25, 75, min-max)	53 (45, 63, 31–70)	52 (44, 59, 10–70)	.132^c^
I have childcare available when I need it			
Agree or slightly agree	37 (80.4)	1660 (76.7)	.550^a^
Disagree or strongly disagree	9 (19.6)	505 (23.3)	

No answers were excluded

*EPDS* Edinburgh Postnatal Depression Scale

^a^Chi-squared test

^b^Fisher’s exact probability test

^c^Mann-Whitney *U* test

Table 5 shows results of logistic regression for maternal depression with associated factors as independent variables. Being in a stepfamily was not associated with maternal depression. However, maternal age was independently associated with maternal depression, with an odds ratio of 0.94 for every 1-year increase in maternal age. Maternal self-perceived health was also associated with maternal depression and so was poor maternal health, where the odds ratio indicated that mothers in poor health were 2.36 times more likely to be depressed than mothers who were not. Stress level was associated with maternal depression, with an odds ratio of 1.67 for every 1-point increase in stress level. Lacking confidence in breastfeeding was associated with maternal depression, with mothers without confidence in breastfeeding 1.83 times more likely to be depressed than mothers with confidence in breastfeeding. Moreover, confidence in the parent role was associated with maternal depression, with mothers who felt a lack of confidence in the parent role 2.49 times more likely to be depressed than mothers who felt confident in the parent role. In addition, number of children was associated with maternal depression, with mothers who had two children being 0.63 times more likely to be depressed than mothers who had one child.

**Table 5:** Result of logistic regression on maternal depression and associated factors

	Odds ratio	95% confidence interval	*p* value
Stepfamily			
No	1.00		
Yes	2.62	0.99–6.91	*.052*
Maternal age (continuous value)	0.94^a^	0.91–0.98	.001
Maternal self-perceived health			
Very good, good, or normal	1.00		
Not so good or poor	2.36	1.29–4.33	.006
Stress score (continuous value)	1.67^b^	1.55–1.81	<.001
I can breastfeed my child as I expect			
Agree or slightly agree	1.00		
Disagree or strongly disagree	1.83	1.28–2.62	.001
I fulfill the parent role well			
Agree or slightly agree	1.00		
Disagree or strongly disagree	2.49	1.76–3.51	<.001
Number of children			
1	1.00		
2	0.63	0.44–0.90	.010
≥3	0.68	0.39–1.19	.173

Non-significant factors other than stepfamily were excluded

^a^Odds ratio for every 1-year increase

^b^Odds ratio for every 1-point increase

## Discussion

### Characteristics of stepfamilies

Results of this study indicated that 2% of all households with infants were stepfamilies. Currently, Japan has no official statistical report on stepfamilies for comparison to determine if our result is larger or smaller than average. According to the 2014 Marriage and Divorce Statistics, 26.4% of marriages between heterosexual couples who married in that year were remarriages. However, the percentage of remarriages with children was unclear. Therefore, we cannot estimate the percentage of stepfamily marriages.

On the other hand, Nishimura [7] reported a 1.8% rate of stepfamilies or their functional equivalents. Inaba [22] also reported a 3.6% rate of stepfamilies or their equivalents. These reported rates are similar to the 2% rate found in the present study. In fact, some have argued that stepfamilies are less socially recognized in Japan due to their relatively small numbers [5]. Findings in our study also indicate that the stepfamily appears to be a family form in the minority.

Our results also identified multifaceted characteristics of stepfamilies. As for basic personal parental information, more parents in stepfamilies, including both mothers and fathers, appear to have not more than a high school education (approximately 60%) compared to parents in non-stepfamilies. This finding of lower parental educational attainment in stepfamilies is consistent with results of Yoda [23], who investigated associations between family structure and child educational expectations.

As for family and financial situations, the rate of families with ≥3 children was higher among stepfamilies (over 50%) than among non-stepfamilies. The rate of families with a first child aged ≥5 years in stepfamilies exceeded 70%, which greatly surpassed the 14.2% rate in non-stepfamilies. Having many children in a household will have a considerable impact on household finances [24]. Educational costs can also become a more serious concern, particularly with respect to older children. In this study, the rate of mothers who felt they lacked economic resources exceeded 60% in stepfamilies, surpassing the 40% rate in non-stepfamily mothers. Low levels of educational attainment among children in stepfamilies have recently been reported in Japan [22], which is consistent with findings reported elsewhere [2]. Thus, financial support for stepfamilies should be considered in child welfare policymaking.

In regard to prenatal maternal and child health, approximately 60% of stepfamily mothers had never participated in childbirth education classes, a much higher rate of non-participation than that of non-stepfamily mothers (about 20%). Further studies are needed to investigate the factors responsible for this low participation rate among stepfamily mothers. Our results suggest that stepfamily mothers did not receive adequate health guidance at medical institutions or local public health institutes during pregnancy.

Whereas only 2.4% of non-stepfamily mothers reported having smoked during pregnancy, 19.6% of stepfamily mothers did so, which is more than eight times the rate of their counterparts. Studies in Western countries have reported that smoking during pregnancy is associated with poverty, lower educational attainment, and social disadvantages such as single parenthood [25]. The present study also found significantly higher rates of inadequate economic resources and lower educational attainment among stepfamily mothers relative to non-stepfamily mothers. Studies outside Japan have reported more smokers among grown children of stepfamily origin than among children of intact families with both birth parents, which were attributed to a higher rate of parental smoking [26]. Thus, stepfamily mothers who smoke during pregnancy should receive appropriate anti-smoking guidance, to protect not only maternal health but also children’s health throughout their lifetime. Given the stepfamily mothers’ low participation in childbirth education classes in our study, individualized approaches might be needed. To that end, changes in certain systems in maternal and child health services might be needed, such as including an optional question on the application form of the Mother and Child Health Handbook that allows stepfamily mothers to safely report their stepfamily status in a way that maintains confidentiality and privacy.

Whereas about 30% of non-stepfamily mothers in this study reported that their latest pregnancy was unplanned, over 50% of stepfamily mothers did so. Unplanned pregnancies raise the risk of unwanted pregnancies, which should be an alert with respect to potential child maltreatment protection [13, 27]. In the event that an unwanted pregnancy is revealed during an individual-approach session with a stepfamily mother, continuous support after childbirth should be provided, and it might be prudent to monitor her childcare situation as well.

### Mental health of stepfamily mothers

Results of this study show that the prevalence of depression among stepfamily mothers is high, about twice that among non-stepfamily mothers. In this study, maternal depression was associated with factors including young age, poor self-perceived health, high stress level, low confidence in breastfeeding, a lack of confidence in the parent role, and number of children. Number of children is considered to be related to the birth experience, and compared with the first childbirth, the second childbirth was associated with lower risk of maternal depression. These findings are consistent with previous studies [20, 21]. In a study on postpartum mental health, Tamaki [28] reported that among factors related to maternal depression, low self-evaluation of the mother role along with low self-esteem were most strongly associated with depressive symptoms. In this study, one of three stepfamily mothers felt they did not adequately fulfill the parent role, and compared to non-stepfamily mothers, more stepfamily mothers lacked confidence in themselves as a parent. The high rate of low self-confidence in the parent role can be considered a backdrop to the high prevalence of depression among stepfamily mothers.

Results of logistic regression analysis did not show an association between stepfamily status and maternal depression, which suggests that stepfamily composition does not necessarily increase the risk of maternal depression. Studies on stepfamilies living overseas have found that stepmothers are more likely to have high rates of depressive symptoms compared to mothers with children of their own [10]. Maternal depression is a widely recognized risk factor for child abuse both domestically and internationally [12, 13, 27], and its deleterious effects on children’s psychological and behavioral development are often reported [29]. Based on previous studies, women with depression that continues a year after childbirth are likely to have entered a depressed state earlier in the postpartum period [30]. Therefore, public health nurses or other professionals should conduct an assessment to identify support needs of stepfamily mothers early in the period.

To this end, building a trusting relationship with stepfamily mothers should take place through individual guidance sessions during pregnancy.

Due to unavoidably complex parent roles in stepfamilies, particularly for stepmothers, previous research reported difficulty in playing a stepparent role and building a stepparent-child relationship [2], which is partially attributable to excessive gender role expectations based on norms of motherly love [8, 19]. In contrast, having low levels of traditional gender role perceptions is reported to function as a protective factor in stepfamily mothers’ mental health [11]. Thus, professionals need to consider the stepfamily mother’s place in her family based on a deep understanding of the difficulties surrounding stepfamily formation. Professionals should also help stepmothers and their husbands understand that they need not be unduly bound by the ideals of standard first-marriage nuclear families and suggest alternatives for stepmothers to become something other than “mother” [24]. It is also important to provide continuous support to mothers with depression and to link the support to a healthcare agency or other professional agency with counseling services as needed.

### Study limitations

A limitation in this study is the participants’ relatively young age due to their being mothers with infants. Also, mean duration of living with a partner was only 4 years as participants were at an earlier stage of stepfamily formation [2]. Therefore, participants in this study might not be representative of all stepfamilies. Although we obtained a relatively high response rate of 75.1%, we cannot exclude the possibility that the response rate of stepfamily mothers was lower than that of non-stepfamily mothers. The most practical means for obtaining more thorough information about stepfamilies is the self-report system at the time of the Mother and Child Health Handbook issuance. Also, this study analyzed factors of maternal depression for all mothers. Therefore, the possibility exists that there are other factors associated with maternal depression peculiar to stepfamily mothers. In this regard, further investigation will be needed.

Nevertheless, this study is the first domestic population-based investigation of stepfamilies in Japan, with a sample composed of all mothers who brought their 3- to 4-month-old infants to a health checkup. Our findings will be useful as a primary source of information to provide better maternal and child health services to stepfamily mothers with infants.

## Conclusions

This study reveals that stepfamilies exhibit many characteristics related to social disadvantage and problems with community-based health care in Japan. Healthcare providers should be aware of stepmothers’ support needs and should put in place a support system for stepfamilies. Moreover, compared with non-stepfamily mothers, stepfamily mothers have a significantly higher prevalence of depression. Also, the high rate of lack of confidence in parenting can be considered the backdrop to a high prevalence of depression among stepfamily mothers, whereas stepfamily composition does not necessarily increase the risk of maternal depression. Therefore, healthcare providers should put in place a system for obtaining more thorough information about stepfamilies and conduct an early assessment to identify their support needs.

## Abbreviation

EPDS: Edinburgh Postnatal Depression Scale
