# Erratum to: Characteristics of stepfamilies and maternal mental health compared with non-stepfamilies in Japan

**DOI:** 10.1186/s12199-017-0665-0

**Published:** 2017-06-16

**Authors:** Masako Sugimoto, Yoshie Yokoyama

**Affiliations:** Department of Public Health Nursing, Osaka City University, 1-5-17 Asahi-machi, Abeno-ku, Osaka, 545-0051 Japan

## Erratum

After the publication of this article [1] it has come to our attention that the ethical approval receipt number of this study was not included in the article. As such, please note that this study [1] was approved by the Ethics Committee of Osaka City University (receipt no. 27–8-1).
